# Diatoms from small ponds and terrestrial habitats in Deserta Grande Island (Madeira Archipelago)

**DOI:** 10.3897/BDJ.9.e59898

**Published:** 2021-02-12

**Authors:** Vítor Gonçalves, Catarina Ritter, Helena Marques, Dinarte Nuno Teixeira, Pedro M. Raposeiro

**Affiliations:** 1 Faculty of Sciences and Technology, University of the Azores, Ponta Delgada, Portugal Faculty of Sciences and Technology, University of the Azores Ponta Delgada Portugal; 2 CIBIO, Research Center in Biodiversity and Genetic Resources, InBIO Associate Laboratory - Azores, Ponta Delgada, Portugal CIBIO, Research Center in Biodiversity and Genetic Resources, InBIO Associate Laboratory - Azores Ponta Delgada Portugal; 3 Instituto das Florestas e Conservação da Natureza IP-RAM, Jardim Botânico da Madeira – Eng. Rui Vieira, Caminho do Meio, Bom Sucesso, Funchal, Portugal Instituto das Florestas e Conservação da Natureza IP-RAM, Jardim Botânico da Madeira – Eng. Rui Vieira, Caminho do Meio, Bom Sucesso Funchal Portugal; 4 Centre for Ecology, Evolution and Environmental Changes, Faculty of Sciences (CE3C), University of Lisbon, Edf. C2, Campo Grande, Lisboa, Portugal Centre for Ecology, Evolution and Environmental Changes, Faculty of Sciences (CE3C), University of Lisbon, Edf. C2, Campo Grande Lisboa Portugal; 5 Laboratory for Integrative Biodiversity Research (LIBRe), Finnish Museum of Natural History, University of Helsinki, Pohjoinen Rautatiekatu, Helsinki, Finland Laboratory for Integrative Biodiversity Research (LIBRe), Finnish Museum of Natural History, University of Helsinki, Pohjoinen Rautatiekatu Helsinki Finland

**Keywords:** Bacillariophyta, Oceanic Islands, freshwater systems, terrestrial systems, Madeira Archipelago, Desertas sub-archipelago

## Abstract

**Background:**

Freshwater diversity, and diatoms in particular, from Desertas Islands (Madeira Archipelago, Portugal) is poorly known, although the Islands are protected and became a Natural Reserve in 1995. During two field expeditions in 2013 and 2014 to Deserta Grande Island, several freshwater and terrestrial habitats were sampled. The analysis of these samples aims to contribute to the biodiversity assessment of the freshwater biota present in Deserta Grande Island. Here, we present the diatom diversity in Deserta Grande Island resulting from that survey. This study contributes to improve the knowledge of Madeira Archipelago freshwater diversity, particularly in the Desertas sub-archipelago.

**New information:**

To our knowledge, we present the first diatom data for the Desertas sub-archipelago. This work resulted in a list of 60 diatom taxa for Deserta Grande, from which 57 were identified to species level. From the 60 new records for Desertas sub-archipelago, 30 of them were also new records for Madeira Archipelago. Several specimens could not be assigned to a known species and may be new diatom species not yet described.

## Introduction

Diatoms (phylum Bacillariophyta Karsten, 1928) are an ubiquitous, highly successful and distinctive group of eukaryotic microalgae, essentially unicellular, which are present in almost every aquatic environment (Round et al. 1990). They are major constituents of benthic and planktic algal communities worldwide in terrestrial, freshwater and marine habitats (Mann and Droop 1996). In addition, the ecological specificity of many diatoms species allows them to be used as environmental indicators (Smol and Stoermer 2010). Freshwater diatom communities have been studied from several continents and remote Oceanic Islands (e.g. Van de Vijver et al. 2002, Bouchard et al. 2004, Grenier et al. 2006, Delgado et al. 2012, Gonçalves et al. 2015, Falasco et al. 2016, Bak et al. 2017).

The Desertas sub-archipelago is formed by three remote small islands, belonging to the Madeira Archipelago. Due to their remoteness, small area, harsh environment and lack of freshwater sources, these Islands remained uninhabited to this day. There has always been interest in the geological setting, fauna and flora from Desertas (Lowe 1868, Lockley 1952, Bannerman 1965, Matias 1984, Neves et al. 1992) and, in 1995, these Islands started to be protected under the Natural Reserve of the Desertas. The entire south terrestrial and marine areas are classified as an Integral Reserve and the north marine area as a Partial Reserve. Due to the high conservation value of these Islands (Neves et al. 1992), fauna and flora have been a matter of study in the last years (e.g. Voigt and Leitner 1998, Nunes 2000, Pires and Neves 2001, Crespo et al. 2013, Crespo et al. 2014, Boieiro et al. 2018, Teixeira et al. 2019); however, to our knowledge, freshwater biota has never been studied. The knowledge of microbial diversity in remote areas with reduced human presence, as are Oceanic Islands, is fundamental for the study of biogeography patterns and meta-community structures amongst microrganisms. Although microorganisms have been considered cosmopolitan, based on the hypothesis that “everything is everywhere, but the environment selects” (Baas Becking 1934), several recent studies show that microorganisms, including diatoms, exhibit biogeographical and macroecological patterns (e.g. Foissner 2006, Martiny et al. 2006, Soininen 2007, Vanormelingen et al. 2008, Verleyen et al. 2009). Diatoms are particularly useful for the study of macroecology conceptual frameworks for microorganisms (Benito et al. 2018), but such studies rely on the existence of large species distribution datasets covering a broad geographical scale (Vanormelingen et al. 2008).

This study presents a taxonomical characterisation of the diatoms found in Deserta Grande freshwaters. We aim to contribute to the current knowledge of diatom diversity and distribution in the Macaronesian Archipelagos and to provide diatom distribution records for regional and global diatom meta-community analysis.

## Project description

### Title

Diatom diversity from Deserta Grande Island (Madeira Archipelago)

### Personnel

Collections were undertaken during the field sampling campaigns in 2013 and 2014 in Deserta Grande Island. The collectors were Pedro Raposeiro and Dinarte Teixeira. Identification was done by Vítor Gonçalves and Helena Marques. Catarina Ritter created the occurrence dataset. The work was supervised by Vitor Gonçalves.

### Study area description

**Figure 1.**
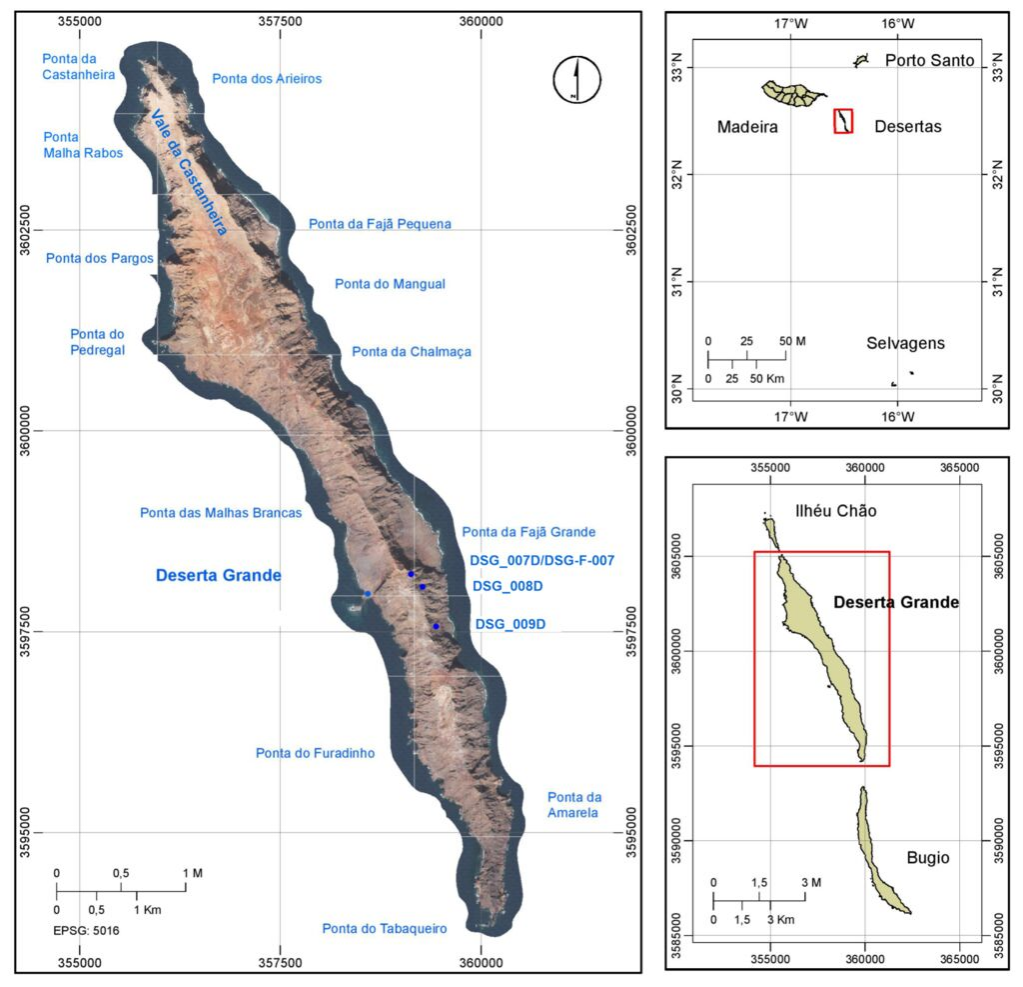
Location of the Madeira Archipelago, the Desertas subarchipelago, Deserta Grande and sampled freshwater habitats.

the Desertas sub-archipelago is formed by three uninhabited small islands belonging to the Madeira Archipelago, located 20 km southeast of Madeira Island (Fig. 1). With an age of 3.6 Ma (Schwarz et al. 2005), the Desertas Islands were connected by a land bridge to Madeira Island during the last glacial period (18,000 years BP) (Brehm et al. 2003). Today, the depth of the sea between Ponta de São Lourenço Peninsula (eastern tip of Madeira Island) and Ilhéu Chão is about 90 m (Geldmacher et al. 2001). Deserta Grande is the largest of the three Islands, with an area of approximately 10 km^2^ and a maximum altitude of 479 m. The Deserta Grande geomorphology is mostly rugged, with very steep slopes, ridges and peaks. The climate is temperate oceanic and the predominant habitats are rocky slopes and small arid flatlands, with sparse vegetation. Freshwater habitats are reduced to temporary streams in Vale da Castanheira and some very small rock pools scattered across the Island. Madeira Archipelago was included as one of the global biodiversity hotspots, together with Azores and the Canary Islands, due to their unique biodiversity (Médail and Quézel 1999, Myers et al. 2000).

## Sampling methods

### Study extent

**Figure 2.**
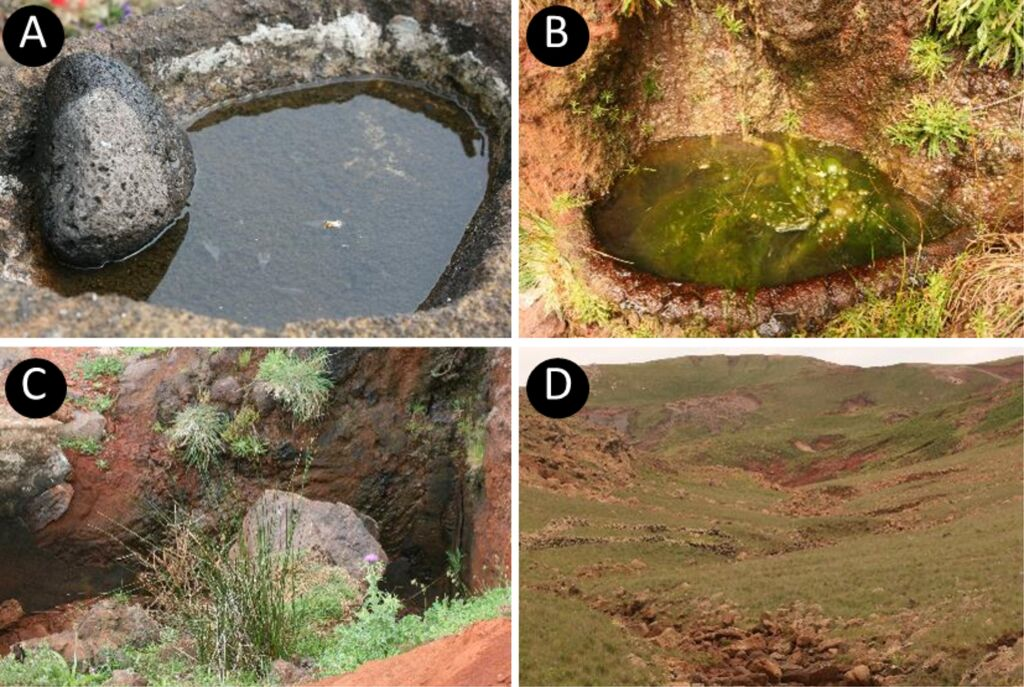
Representative freshwater habitats in Deserta Grande: **A.** water reservoir; **B, C.** natural pools; **D.** temporary stream in Vale da Castanheira (photos by Pedro Raposeiro).

Freshwater habitats ranging from water reservoir, natural pools and temporary streams were sampled during two field campaigns in 2013 and 2014 in Deserta Grande Island (Fig. 2). Five samples were collected at four sites (Table 1).

### Sampling description

Diatom samples were collected in 2013 and 2014 by filtering water or by brushing the bottom and walls of the pools or stream bed. With the help of a toothbrush to remove the biofilm, the sample was placed into a tray with a little water and the resulting suspension was collected in a plastic container, fixed with alcohol and stored prior to analysis. Samples were treated with warm nitric acid and mounted with Naphrax©, according to European and national recommendations (Kelly et al. 1998, INAG 2008). Diatom slides were examined under differential interference contrast light microscopy using a ZEISS AXIOIMAGE A1 microscope with an immersion Plan-Apochromat 100x objective (NA 1.40).

### Quality control

Species identification was made by trained taxonomists with the help of European diatom floras (Krammer and Lange-Bertalot 1986, Krammer and Lange-Bertalot 1988, Krammer and Lange-Bertalot 1991, Krammer and Lange-Bertalot 2000, Krammer and Lange-Bertalot 2002). Diatom morphometric features were determined on photomicrographs taken with a ZEISS AxioCam MRc5 attached to the microscope with the aid of image analysis software (ZEISS Axiovision SE64). To determine species relative abundance, at least 400 valves were counted in each sample.

### Step description

The data have been published as a Darwin Core Archive (DwC-A), which is a standardised format for sharing biodiversity data as a set of one or more data tables. The core data table contains 149 occurrences with 60 taxa (taxonID) (Gonçalves et al. 2020).

## Geographic coverage

### Description

Deserta Grande, Desertas sub-archipelago, Madeira Archipelago, Macaronesia, Portugal.

### Coordinates

32.396 and 32.604 Latitude; -16.563 and -16.449 Longitude.

## Taxonomic coverage

### Description

All diatoms were identified to genus or species level. A total of 60 taxa were found, from which 57 were identified to species and three to genus level. The species found belong to 22 families, 13 orders, five subclasses and three classes (Table 2).

## Traits coverage

### Data coverage of traits

PLEASE FILL IN TRAIT INFORMATION HERE

## Temporal coverage

### Notes

2013-09-18,2014-04-14

## Usage licence

### Usage licence

Open Data Commons Attribution License

### IP rights notes

This work is licensed under a Creative Commons Attribution (CC-BY) 4.0 License.

## Data resources

### Data package title

Diatoms from Deserta Grande (Madeira Archipelago, Portugal)

### Resource link

http://ipt.gbif.pt/ipt/resource?r=diatdes

### Alternative identifiers

https://www.gbif.org/dataset/03dfa40e-3887-4648-8fc2-e72e0bd09fbd

### Number of data sets

1

### Data set 1.

#### Data set name

Diatoms from Deserta Grande (Madeira Archipelago, Portugal)

#### Data format

Darwin Core

#### Number of columns

29

#### Data format version

1.5

#### Description

This dataset presents the first data on the distribution of freshwater diatoms in Deserta Grande Island (Madeira Archipelago). The dataset has been published as a Darwin Core Archive (DwC-A), which is a standardised format for sharing biodiversity data as a set of one or more data tables. The core data table contains five events (eventID), 149 occurrences (occurrenceID) with 60 taxa (taxonID). The number of records in the data table is illustrated in the IPT link. This IPT archives the data and thus serves as the data repository. The data and resource metadata are available for downloading in the downloads section.

**Data set 1.** 

Column label	Column description
type	The nature of the resource.
basisOfRecord	The specific nature of the data record.
occurrenceID	Identifier of the record, coded as a global unique identifier.
eventID	Identifier of the event, unique for the dataset.
eventDate	Time interval when the event occurred.
locality	Name of the locality where the event occurred.
continent	Continent of the sampling site.
islandGroup	Island group of the sampling site.
island	Island from the Island Group of the sampling site.
country	Country of the sampling site.
countrycode	Code of the country where the event occurred.
scientificNameAuthorship	The authorship information for the scientificName.
coordinateUncertaintyInMeters	The indicator for the accuracy of the coordinate location in meters, described as the radius of a circle around the stated point location.
decimalLatitude	The geographic latitude of the sampling site.
decimalLongitude	The geographic longitude of the sampling site.
geodeticDatum	The spatial reference system upon which the geographic coordinates are based.
taxonID	The identifier for the set of taxon information (data associated with the Taxon class). Specific identifier to the dataset.
scientificName	The name with authorship applied on the first identification of the specimen.
acceptedNameUsage	The specimen accepted name, with authorship.
kingdom	Kingdom name.
phylum	Phylum name.
class	Class name.
order	Order name.
family	Family name.
genus	Genus name.
specificEpithet	The name of the first or species epithet of the scientificName.
infraspecificEpithet	The name of the lowest or terminal infraspecific epithet of the scientificName, excluding any rank designation.
taxonRank	The taxonomic rank of the most specific name in the scientificName.
Municipality	Name of the municipality where the event occurred.

## Additional information

### Analysis

**Figure 3.**
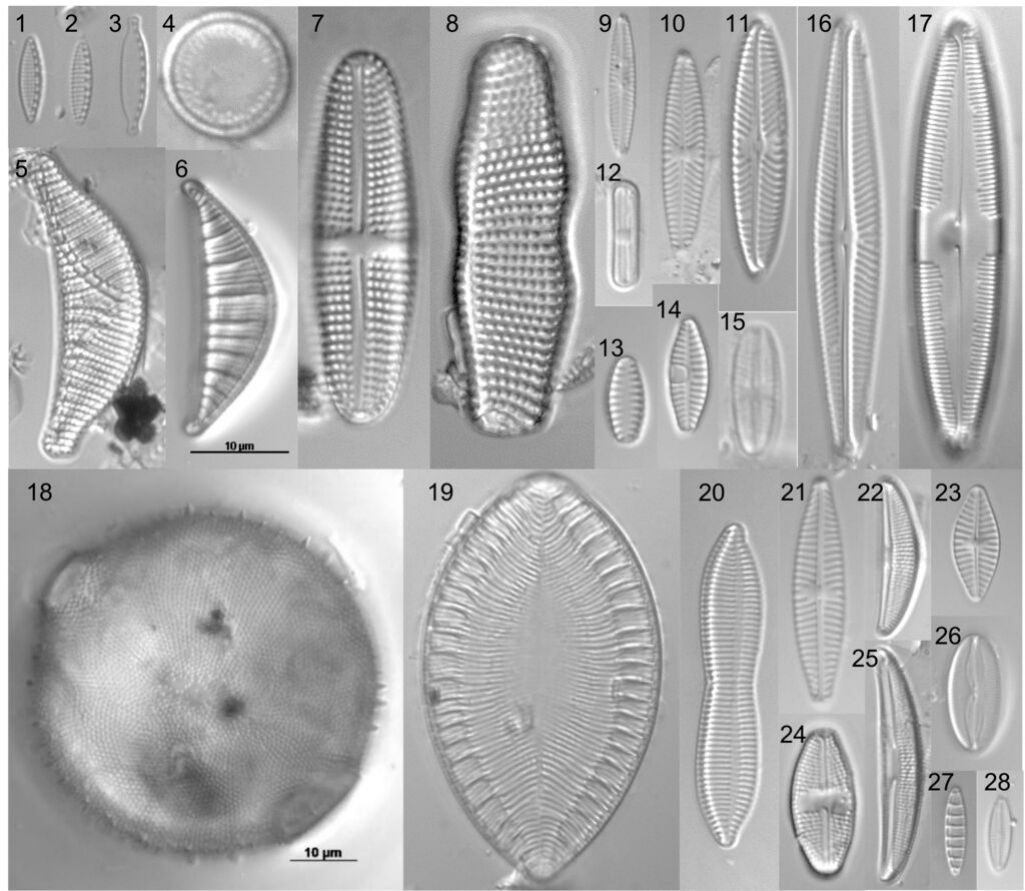
Some of the most common and abundant diatoms in Deserta Grande Island: 1- *Nitzschia inconspicua*; 2- *Nitzschia valdestriata*; 3- *Nitzschia microcephala*; 4- Aulacoseira cf. perglabra 5- *Epithemia sorex*; 6- *Epithemia operculata*; 7- Achnanthes brevipes var. intermedia; 8- *Achnanthes coarctata*; 9- *Navicula vilaplanii*; 10- *Navicula cari*; 11- *Navicula* sp.1; 12- *Humidophila contenta*; 13- *Pseudostaurosira* sp.1; 14- *Planothidium frequentissimum*; 15- *Sellaphora nigri*; 16- *Navicula radiosafallax*; 17- *Caloneis molaris*; 18- *Pleurosira laevis*; 19- *Surirella ovalis*; 20- *Tryblionella apiculata* 21- *Navicula veneta*; 22- *Halamphora veneta*; 23- *Planothidium delicatulum*; 24- *Luticola mutica*; 25- *Halamphora paraveneta*; 26- *Fallacia pygmaea*; 27- *Denticula subtilis*; 28- *Pseudofallacia monoculata*. Scale bar on picture 6 applies for all images, except picture 18.

The most common species were *Achnanthidium minutissimum* (Kützing) Czarnecki, *Denticula subtilis* Grunow, *Halamphora veneta* (Kützing) Levkov, *Humidophila contenta* (Grunow) Lowe, Kociolek, J.R.Johansen, Van de Vijver, Lange-Bertalot & Kopalová, *Navicula cari* Ehrenberg, *Navicula veneta* Kützing, *Nitzschia inconspicua* Grunow, *Planothidium delicatulum* (Kützing) Round & Bukhtiyarova, *Planothidium frequentissimum* (Lange-Bertalot) Lange-Bertalot, *Pleurosira laevis* (Ehrenberg) Compère and *Epithemia operculata* (C.Agardh) Ruck & Nakov (Fig. 3). These eleven species occurred in all five studied samples. *Achnanthes coarctata* (Brébisson ex W.Smith) Grunow, *Nitzschia valdestriata* Aleem & Hustedt, *Planothidium lanceolatum* (Brébisson ex Kützing) Lange-Bertalot and *Sellaphora nigri* (De Notaris) C.E.Wetzel & L.Ector were also very common and appeared in four of the five samples (Suppl. material 1).

Species that occurred in just one sample with less than 1% abundance were considered rare (Suppl. material 1). Amongst these were included *Encyonema silesiacum* (Bleisch) D.G.Mann, *Epithemia adnata* (Kützing) Brébisson, *Fallacia pygmaea* (Kützing) Stickle & D.G.Mann, *Eunotia exigua* (Brébisson ex Kützing) Rabenhorst, *Fragilaria capucina* Desmazières, *Fragilaria vaucheriae* (Kützing) J.B.Petersen, *Frustulia rhomboides* (Ehrenberg) De Toni, *Gomphonema olivaceum* (Hornemann) Brébisson, *Navicula gregaria* Donkin, *Navicula metareichardtiana* Lange-Bertalot & Kusber, *Nitzschia frustulum* (Kützing) Grunow, *Nitzschia palea* (Kützing) W.Smith, *Nitzschia perspicua Cholnoky*, *Nitzschia soratensis* E.A.Morales & M.L.Vis, *Rhoicosphenia abbreviata* (C.Agardh) Lange-Bertalot, *Rhopalodia rupestris* (W.Smith) Krammer, *Sellaphora saugerresii* (Desmazières) C.E.Wetzel & D.G.Mann, *Tabellaria flocculosa* (Roth) Kützing and *Ulnaria biceps* (Kützing) Compère and one unidentified species from the genus *Cocconeis* Ehrenberg.

From the 60 taxa found in Deserta Grande, 30 of them were new records for the Madeira Archipelago. These belonged to 15 families in eight orders. Most of the new records belonged to the orders Naviculales (16 species) and Bacillariales (six species).

The diatom flora of Deserta Grande is mainly constituted by cosmopolitan species, but some taxa were impossible to assign to a known species and may belong to undescribed species. The possible existence of endemic species for the Island of Deserta Grande, in particular and the Madeira Archipelago, in general, would not be surprising considering the volcanic origin and remoteness of these Islands, which favours speciation (Whittaker et al. 2008). High levels of island and regional endemisms were found in other Oceanic Islands in the South Atlantic. For instance, aproximately 33% species found in the Falkland Islands were considered island or regional endemisms (Flower 2005), whereas Carter (1966) described 55 new species for Tristan da Cunha Archipelago. More recently, several new species were described from South Atlantic islands, such as from Ascension Island (Van de Vijver et al. 2019, Van de Vijver et al. 2018), Deception Island (Van de Vijver and Mataloni 2008), Falkland Islands (Juttner et al. 2018) and Gough Island (Van de Vijver and Kopolova 2008). Similarly, in the North Atlantic, several endemic diatoms were described in the Oceanic Islands of Madeira (Lange-Bertalot 1993, Lange-Bertalot et al. 2011) and Azores (Delgado et al. in press). Thus, a more thorough survey and more detailed analysis of the fine structure of the frustule with a scanning electron microscope in the future is needed to fully describe the diversity and distribution of diatoms in Desertas Islands and this may result in the description of many new taxa.

## Supplementary Material

0CAD3AD7-640C-58D9-927F-804DD98A23B7 10.3897/BDJ.9.e59898.suppl1 Supplementary material 1 Relative diatom abundances in the studied samples from Deserta Grande Data type Abundances and occurrences File: oo_484297.xlsx https://binary.pensoft.net/file/484297 Vítor Gonçalves, Catarina Ritter, Helena Marques, Dinarte Nuno Teixeira and Pedro M. Raposeiro

## Figures and Tables

**Table 1.** Samples code, date and location of the sampling sites in Deserta Grande Island.

Sample Code	Sampling date	Locality	Latitude (ºN) / Longitude (ºW)	Altitude (m)
DSG_2013	2013-09-18	Baixio (close to Doca)	32,513042, -16,50931	20
DSG-F- 007	2014-04-14	Close to Ponta da Fajã Grande	32,51778, -16,50589	217
DSG-007D	2014-04-14	Close to Ponta da Fajã Grande	32,51778, -16,50589	217
DSG-008D	2014-04-14	Fajã Grande	32,516021, -16,50490	213
DSG-009D	2014-04-14	C. da Doca	32,507762, -16,50111	194

**Table 2.** Taxonomic coverage of the data. The number of genera and species included in each higher taxon is presented.

Rank	Scientific name	Number of genera	Number of species
Kingdom	Chromista	34	60
Phylum	Bacillariophyta	34	60
Class	Bacillariophyceae	32	58
Subclass	Bacillariophycidae	26	51
Order	Bacillariales	4	14
Family	Bacillariaceae	4	14
Order	Cocconeidales	4	5
Family	Achnanthidiaceae, Cocconeidaceae	4	5
Order	Cymbellales	3	3
Family	Gomphonemataceae, Rhoicospheniaceae	3	3
Order	Mastogloiales	1	2
Family	Achnanthaceae	1	2
Order	Naviculales	12	22
Family	Naviculaceae, Amphipleuraceae, Brachysiraceae, Diadesmidaceae, Pinnulariaceae, Sellaphoraceae, Naviculales incertae sedis	12	22
Order	Rhopalodiales	2	4
Family	Rhopalodiaceae	2	4
Order	Surirellales	1	1
Family	Surirellaceae	1	1
Subclass	Eunotiophycidae	1	1
Order	Eunotiales	1	1
Family	Eunotiaceae	1	1
Subclass	Fragilariophycidae	5	6
Order	Fragilariales	2	3
Family	Fragilariaceae, Staurosiraceae	2	3
Order	Licmophorales	1	1
Family	Ulnariaceae	1	1
Order	Tabellariales	2	2
Family	Tabellariaceae	2	2
Class	Mediophyceae	1	1
Subclass	Thalassiosirophycidae	1	1
Order	Eupodiscales	1	1
Family	Eupodiscaceae	1	1
Class	Coscinodiscophyceae	1	1
Subclass	Coscinodiscophycidae	1	1
Order	Aulacoseirales	1	1
Family	Aulacoseiraceae	1	1
